# Hemodynamic Alteration in Aortic Valve Stenosis: CFD Insights from Leaflet-Resolved Models

**DOI:** 10.3390/bioengineering12101029

**Published:** 2025-09-26

**Authors:** Mashrur Muntasir Nuhash, Victor K. Lai, Ruihang Zhang

**Affiliations:** 1 Department of Mechanical and Industrial Engineering, University of Minnesota Duluth, Duluth, MN 55812, USA; nuhas001@d.umn.edu; 2 Department of Chemical Engineering, University of Minnesota Duluth, Duluth, MN 55812, USA; laix0066@d.umn.edu

**Keywords:** aortic stenosis, computational fluid dynamics, hemodynamics, aortic arch, helicity, pressure loss coefficient

## Abstract

Aortic valve stenosis, is a prevalent cardiovascular disease, narrows the valve orifice and restricts blood flow, resulting in abnormal high velocities and shear stresses. The progression of these hemodynamic abnormalities and their link with stenosis severity remain incompletely understood, which are critical for early detection and intervention. Computational Fluid Dynamics (CFD) was employed to characterize aortic hemodynamics across healthy, mild, moderate, and severe stenosis using a 3D steady-state model with idealized leaflet geometries. Key flow parameters, including velocity distribution, wall shear stress (WSS), pressure loss coefficient, and helicity, were evaluated. Results show a non-linear increase in velocity and WSS with stenosis severity, with peak jet velocities of 1.08, 1.82, 2.73, and 4.7 m/s and peak WSS of 11, 35, 80, and 122 Pa at the aortic arch, respectively. Severe stenosis produced a highly eccentric jet along the anterior of aortic arch, accompanied by a narrower jet, increased turbulence intensity and expanded recirculation zones. A significant increase in helicity and pressure loss coefficient was also observed for higher stenosis severities. These findings highlight the influence of valve leaflets on aortic flow dynamics, providing physiologically relevant insights into stenosis-induced mechanical stresses that may drive endothelial dysfunction and support earlier detection of disease progression.

## 1. Introduction

Aortic valve stenosis is one of the most prevalent valvular diseases worldwide, affecting around 2% of people over the age of 60 [1]. It is a progressive disease that the valve leaflets become thick and stiff, leading to a narrowing of the valve orifice area. This obstruction results in restricted blood flow and an elevated pressure gradient to pump blood through the valve, which causes hemodynamic abnormalities [2]. The onset of this disease starts from the structural damage around the leaflets. Continuous abnormal shear stress and turbulence can damage endothelial cell function and allow lipoproteins to invade the valve [3]. These lipoproteins can become oxidized and cause inflammation [4]. Further progression of this disease causes changes in the valvular interstitial cells, leading to the thickening of the valves and calcification [5,6]. Stiff and calcified valves narrow orifice areas at the systole, forcing the heart to work harder which can potentially lead to heart failure [7]. The eccentricity and high kinetic energy of the jet impinging from the valve orifice can enhance localized wall shear stresses (WSS) and weaken the overall structure of the walls around the arch [8]. However, as a progressive disease, not all patients develop apparent symptoms at its early stages. For instance, even for some older patients, the initial signs can be only limited to a decrease in exercise tolerance [9]. Moreover, researchers have found that the progression rate of this disease surges with increasing severity, with severe stenosis being particularly critical [10]. However, it’s possible that a patient may remain asymptotic for a long time, while previous studies have reported that once calcification develops, over 50% of untreated patients may result in mortality [2,11]. Therefore, early detection is crucial, utilizing appropriate markers and predictors for both hemodynamic and anatomical abnormalities.

A variety of methods has been employed to investigate the hemodynamics abnormalities induced by aortic stenosis. Among them, 4D MRI is a promising approach for predicting turbulence in the aorta; however, its relatively low spatial and temporal resolution may reduce its accuracy and affect the reliability of the results [12]. Subsequently, in-vitro experimental visualization techniques such as particle image velocimetry (PIV) and laser Doppler velocimetry (LDV) are commonly utilized to explore valve hemodynamics as they provide better spatial and temporal resolution [13,14]. Yap et al. [14] investigated the shear stress distribution on the aortic valve using LDV in an in vitro setup, under varying cardiac outputs. The researchers quantified peak systolic shear stress of 21 dyn/cm^2^ at the leaflets, which significantly reduces to 4 dyn/cm^2^ at diastole. They concluded that oscillatory shear stress could play a significant role in triggering valve calcification. Büsen et al. [15] conducted a PIV experiment to understand the influence of age-related compliance effects on hemodynamics. They found that reducing compliance from 1.82 to 0.62 × 10^−3^ mmHg^−1^ led to a 56% increase in early systolic velocities and a marked 122% rise in the rate of pressure change. Zhang et al. [16] also conducted a PIV study of a compliant aortic root to characterize its impact on flow dynamics, under variable cardiac outputs. The authors found that peak turbulence kinetic energy (TKE) arises shortly after systole, which also coincides with maximum structural deformation. Researchers also utilized these methods to study stenotic aortic valves. Ding et al. [17] experimentally investigated how the stenosis level influences the onset of turbulence and shear stress distribution. They reported that turbulence intensifies with stenosis levels, and 75% stenosis enables the jet to reach the opposite wall, while significantly increasing the oscillatory shear index (OSI) to 0.3. Zhang et al. [18] performed another PIV study on a stenotic prosthetic aortic valve and revealed that severe stenosis resulted in approximately a twofold increase in peak jet velocity, threefold increase in TKE, more eccentric jet profiles, and elevated Reynolds shear stress (RSS).

Numerical methods such as computational fluid dynamics (CFD), serve as a powerful tool which can complement these experimental methods. CFD can generate predictive results relatively quickly and these simulations are easily customizable for patient-specific geometries [19]. These advantages make CFD a valuable tool for the early detection of the disease, especially when used in conjunction with diagnoses from medical professionals. Subsequently, CFD has been extensively used in modeling the behavior of blood flow inside the aorta, utilizing either patient-specific or idealized geometries [12,19,20,21,22,23,24]. However, due to the complex movement of valve leaflets during the cardiac cycle, CFD is often coupled with fluid–structure interaction (FSI) models [25]. Armin et al. [26] successfully captured the movement of stenotic leaflets and related hemodynamics in a 2D aortic valve model. The researchers coupled two modules in ANSYS (Fluent and Mechanical) to predict the deformation of the leaflets for healthy, calcified, and severely calcified valves. Other studies focused on reducing the time constraint and complexity of such FSI models [27,28]. To reduce the computational demands of full FSI models, researchers often develop steady-state CFD models focusing on peak systole with variable effective orifice areas to replicate different levels of stenosis. Jhun et al. [29] investigated the fluid dynamics of blood in mild, moderate, and severe stenosis using a Large Eddy Simulation (LES) model. The geometry consisted of an orifice with a variable diameter and an idealized aortic arch. The authors studied velocity distribution, WSS, and Reynolds Shear Stress (RSS) and found linear relationships between maximum velocity and WSS with stenosis level, but non-linear for RSS. In a different analysis, Zhu et al. [30] utilized Direct Numerical Simulation (DNS) to evaluate the impact of stenosis level on the generation of vortices in the aortic arch using a simplified model of aortic stenosis. They concluded that the anterior arch with maximum pressure fluctuations may be the source of heart murmurs. Another study investigated the change in velocity and WSS due to stenosis in a 2D model of the valve [31], with results indicating significant variations in WSS and velocity profiles across different stenosis severities.

Another important and interesting aspect is hemodynamic influences on valve leaflets, particularly how WSS affects their biomechanical environment and potentially contributes to disease initiation. In a PIV experiment, Moore et al. [32] demonstrated that the inclusion of coronary outflow changes the hemodynamics, augmenting the peak WSS at the leaflet base to 1.2 Pa, which is three times higher than that in the non-coronary sinus. Additionally, the non-coronary sinus experienced more OSI, which may make it more vulnerable to calcification. Cao et al. [33] conducted a 3D FSI analysis of the aortic valve to explore the abnormal WSS experienced by the leaflets. Their analysis revealed that the ventricularis surface experienced lower OSI compared to the fibrosa surface, and the highest WSS magnitude was observed at the base of the leaflets, followed by the belly and the tip. In a subsequent study, they found that incorporating coronary flow further increased WSS on the fibrosa and decreased OSI, particularly in the belly and tip regions [34]. In sum, these studies underscore the critical role of local hemodynamic forces such as WSS and OSI in shaping leaflet behavior and possibly driving the early onset of valvular disease.

So far, most steady-state CFD models of aortic stenosis use simplified orifice geometries, neglecting the anatomical features of valve leaflets that critically influence hemodynamics. This omission limits the ability to capture key flow behaviors such as jet eccentricity, impingement patterns, and WSS, all of which vary with stenosis severity and contribute to disease progression. Including leaflet geometries allows for localized assessment of biomechanical forces on the valve surface, particularly WSS which is strongly related to calcification and structural remodeling. However, the combined influence of leaflet shape and stenosis level on both downstream flow and surface-level shear stress remains poorly understood. Thus, this study aims to address that gap by developing a steady-state 3D CFD model with idealized leaflets representing conditions from healthy to severe stenosis. The model is used to evaluate the effects of leaflet inclusion on velocity distribution, helicity, pressure loss, and leaflet WSS to better characterize the hemodynamic environment associated with disease development.

## 2. Methods

### 2.1. Physical Model and Boundary Conditions

This study presents a three-dimensional (3D), steady-state CFD model of the aorta and valve. The aortic arch is idealized and modelled as a curved pipe with a 180° bend, and the aortic valve leaflet geometry is represented at peak systole, corresponding to the phase of the maximum effective orifice area opening. The severities of aortic stenosis are generated by modifying the valve orifice area, as prescribed by the American College of Cardiology/American Heart Association [35]. The categories of stenosis levels used in this study are presented in Table 1. To compare the impact of stenosis level on the hemodynamics of the aorta, a healthy valve is also designed with an aperture area of 3.15 cm^2^, followed by mild, moderate, and severe stenotic severities with areas of 1.85 cm^2^, 1.25 cm^2^ and 0.85 cm^2^, respectively.

**Figure 1:**
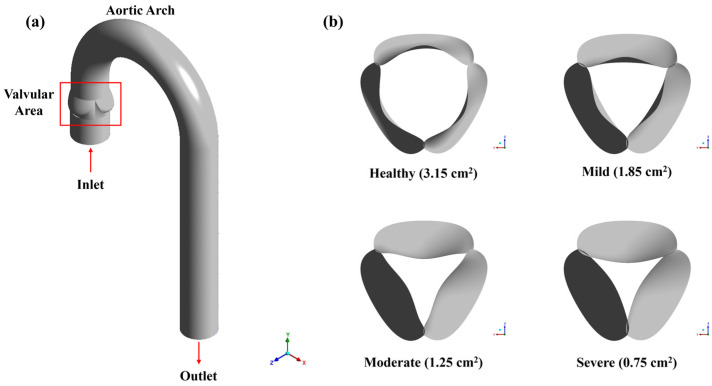
(**a**) The geometry of the computational domain and (**b**) the leaflet shapes and area corresponding to different stenosis levels.

The geometry of the computational domain, along with the leaflet geometry and corresponding aperture area, are displayed in Figure 1. The diameter of the domain upstream of the valvular area is 28.5 mm, while the downstream region, consisting of the aortic arch, maintains a uniform diameter of 25 mm. Jhun et al. suggested increasing the length of the downstream region by 20 times the aortic diameter to eliminate the influence of the outlet on the simulation results [29]. A model with insufficient downstream length can result in reverse flow at the outlet, leading to an artificial pressure gradient in that region. This can introduce numerical instabilities and negatively impact the convergence of the simulation. However, in the case of the current model, reverse flow is not observed with a downstream length of 150 mm, 8 times the aortic diameter, which is used in further calculations to reduce element number. Figure 1a represents the boundary condition of the numerical model. At the inlet, a non-pulsatile mass flow of 0.315 kg·s^−1^ is prescribed at the streamwise z-direction, with a plug velocity profile as suggested by previously published research [26]. Jhun et al. [29] suggested a mass flow rate of 0.315 kg·s^−1^ (18 LPM) as a conservative estimate. Based on the severe stenosis thresholds in Table 1 and the continuity equation, the peak flow can reach up to 24 LPM but may drop to around 17 LPM if the valve area decreases to 0.75 cm^2^. Therefore, 18 LPM was chosen as a balanced value that reflects high jet velocity while keeping the simulation realistic [29]. A pressure outlet condition is employed at the outlet, while the no-slip and rigid condition is applied at the walls. The density of blood is kept at 1050 kg/m^3^. Blood was assumed to be Newtonian with a constant blood viscosity of 0.0035 Pa·s since published literature has shown that this Newtonian assumption does not affect the results significantly in larger arteries such as the aorta [36].

### 2.2. Governing Equations

The continuity and Navier–Stokes equations are used to capture the hemodynamics of both healthy and stenotic aortic valves.

Continuity Equation [37]:

(1) $$\frac{\partial \left(\rho u_{i}\right)}{\partial x_{i}}=0$$

Momentum Equation [37]:

(2) $$\frac{\partial }{\partial x_{j}}\left(\rho u_{i}u_{j}\right)=-\frac{\partial p}{\partial x_{i}}+\frac{\partial }{\partial x_{j}}\left\{\mu \left[\frac{\partial u_{i}}{\partial x_{j}}-\bar{\rho u_{i}^{\prime }u_{j}^{\prime }}\right]\right\}$$

Here, ρ is the fluid density (kg/m^3^), the fluid velocity is u (m/s), p indicates the fluid pressure (Pa), velocity fluctuation is $u_{i}^{\prime }$, and μ indicates the dynamic viscosity of the working fluid (Pa·s). To completely model turbulent flows, a Reynold term is considered using Reynolds averaged methods, which is $\bar{\rho u_{i}^{\prime }u_{j}^{\prime }}$. It can be explained via the Boussinesq hypothesis as:

(3) $$-\bar{\rho u_{i}^{\prime }u_{j}^{\prime }}=\mu_{t}\left[\frac{\partial u_{i}}{\partial x_{j}}+\frac{\partial u_{j}}{\partial x_{i}}\right]-\frac{2}{3}\rho k\delta_{ij}-\frac{2}{3}\mu_{t}\frac{\partial u_{k}}{\partial x_{k}}\delta_{ij}$$

where $k=1/2\bar{u_{i}^{\prime }u_{j}^{\prime }}$ is the turbulent kinetic energy (m^2^/s^2^), $\delta_{ij}$ is the Kronecker delta, and $\mu_{t}$ depicts the turbulent viscosity (Pa·s).

The k-ω SST turbulence model was employed in this study because of its demonstrated performance in simulating large arteries [38], where k and ω depict the turbulent kinetic energy and the diffusion of turbulent energy, respectively.

### 2.3. Mesh Sensitivity Analysis and Numerical Methods

**Figure 2:**
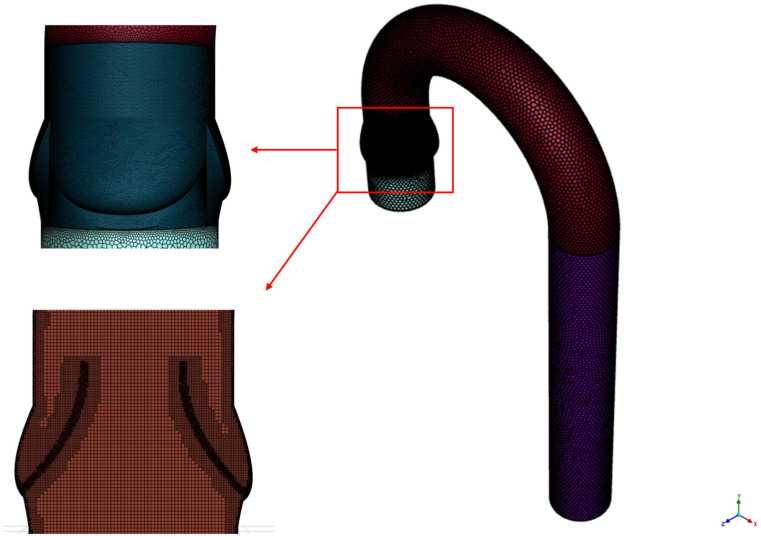
The mesh distribution of the computational domain. The concentrated mesh around the valvular area is highlighted, also depicting the cell layout at volume.

Figure 2 shows the generated grid for the computational domain. 2.4 million polyhexcore cells were generated to discretize the domain. As illustrated in the highlighted section of Figure 2, cells are concentrated in the valvular area and leaflets since they are subjected to a higher velocity gradient. The figure also shows the generated hexagonal volume cells, which significantly reduces the overall number of elements in the computational domain compared to the conventional meshing method. The polyhexcore meshing method generates poly-meshes on the surface and fills the interior volume with hex-core cells, which has been reported to reduce simulation time by up to 50% [39]. Utilizing buffer layers enhances mesh customization as observed near the leaflets in the volume mesh. Overall, the mesh near the walls was refined to ensure y+ ≤ 1, achieving better wall resolution. The wall y+ is a dimensionless distance normal to the wall used to evaluate the resolution of mesh near the wall. To accurately capture the viscous sublayer, y^+^ ≤ 1 is recommended for the k-ω SST turbulence model [40].

A grid sensitivity analysis was conducted using the healthy aortic valve to determine the optimum number of meshes. For the sensitivity analysis, a uniform mass flow of 0.315 kg/s was prescribed at the inlet, with a pressure outlet condition at the exit. A total of 4 element numbers consisting of 0.7, 1.2, 2.4, and 4.4 million cells were considered for this grid independence study. Table 2 depicts the average WSS magnitude at the valve surface for moderate stenosis and a comparison of the different mesh sizes. It can be observed that the WSS for 2.4 million and 4.4 million cells are 2.361 and 2.347, respectively. Since there is a minimal deviation between them (less than 1%), the smaller cell size is chosen for the remainder of this study.

These 3D CFD model simulations were performed using the finite volume-based solver in Ansys Fluent 2023 R2 (Ansys, Inc., Canonsburg, PA, USA). The SIMPLE method was used for pressure velocity coupling, while the second-order upwind scheme was utilized for the momentum equations. The convergence criteria were set to 10^−4^ and 10^−6^ for the continuity equation and momentum equations, respectively.

## 3. Results

In this numerical study, a 3D CFD model with steady-state assumption has been constructed using Ansys fluent to evaluate the impact of aortic stenosis on the hemodynamics of the aortic arch. In the following section, at first, the accuracy of the numerical model is established by comparing the results with an experimental study. Then the variation in hemodynamics is analyzed by comparing the velocity distribution and magnitude, helicity, and WSS at the peak systole phase for various stenosis levels.

### 3.1. Validation

The simulation results were compared with previously published experimental and numerical data to validate the current model. Specifically, the velocity distribution of a moderate aortic stenosis model was validated by comparison with the corresponding experimental measurements of Jhun et al. [29]. The geometry for this validation consists of an upstream domain with a diameter of 38 mm and a downstream diameter of 20 mm. The valve leaflets are replaced by an orifice of 10 mm diameter to replicate moderate stenosis. A mass flow rate of 0.315 kg·s^−1^ (18 LPM) is applied at the inlet while a pressure outlet condition is prescribed for the exit. The k-ω SST turbulence model has been used to resolve turbulence.

**Figure 3:**
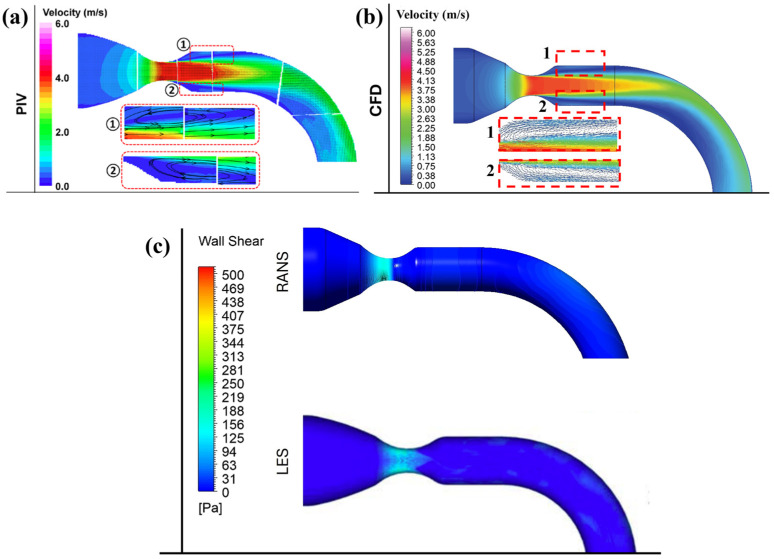
Validation of velocity and WSS distribution, (**a**) PIV results from Jhun et al. [29] and (**b**) the current numerical mode and (**c**) validation of WSS of the current RANS simulation with the LES model of Jhun et al. [29]. Subfigures (**1**) and (**2**) depict the recirculation region downstream of the valve, experimental PIV results [29] and the present CFD model, respectively.

As illustrated in Figure 3a,b, a reasonable agreement between the CFD-generated velocity field and the PIV model is observed. The numerical model correctly captures the distribution of jet velocity and the recirculation zone as shown in the highlighted region of Figure 3b. The maximum velocity depicted by the numerical model is 4.12 m/s compared to 4.2 m/s of the PIV data. The wall shear stress (WSS) distribution in Figure 3c also shows consistency between the current Reynolds-Averaged Navier–Stokes model (RANS) and Jhun et al.’s LES model [29]. Table 3 provides quantitative comparisons between experimental measurements and the current numerical model.

### 3.2. Impact of Aortic Stenosis on Aortic Arch Velocity Profile

**Figure 4:**
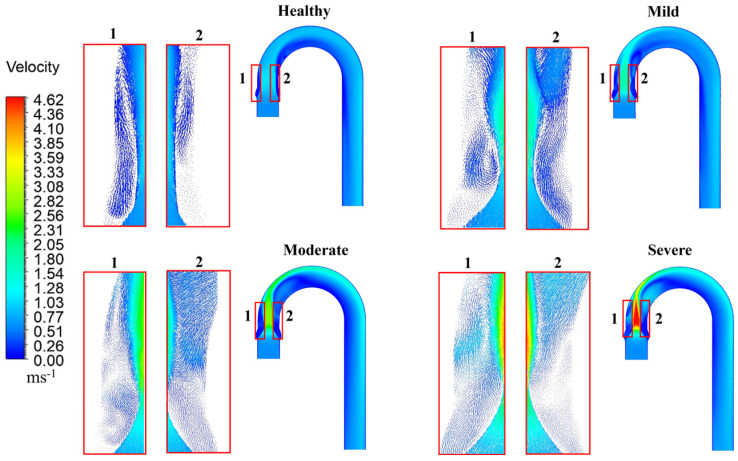
The hemodynamics of the aorta of healthy valves, mild, moderate, and severe stenotic valves, and the recirculation zones are highlighted in the red boxes. Subfigures (**1**) and (**2**) in each scenario indicate the zoom-in region of the simulation results.

Figure 4 illustrates the flow pattern in the aortic arch, highlighting the variation of velocity distribution impacted by the severity of stenosis. As shown, a high-velocity jet emerges just downstream of the valve exit, which travels through the ascending aorta and impinges on the anterior wall of the arch. The high-velocity magnitude of the jet core generates significant recirculation zones in the ascending aorta, as highlighted in the figure. Similarly, flow separation and recirculation are also observed along the anterior wall of the aortic arch. The peak velocity magnitude increases with the severity of stenosis, measuring 1.08, 1.82, 2.73 and 4.7 m/s for the healthy valve, and for the mild, moderate, and severely stenotic valves, respectively.

Moreover, in Figure 4, the healthy aortic valve exhibits a stable and uniform flow distribution throughout the aortic arch. The jet remains steady in the ascending aorta, generating a symmetric recirculation region adjacent to the jet core. Due to leaflet contraction, the jet core gets narrowed with a sharp increase in the velocity magnitude in the case of mild stenosis. As a result, the recirculation region expands in width and becomes more turbulent and asymmetric. For both healthy and mildly stenotic valves, the jet impinges on the arch wall and travels along the anterior side of the aortic arch toward the descending aorta, where it gradually dissipates and becomes more uniform. Such uniformity is not observed in the case of moderate and severe stenosis, where the flow remains highly skewed toward the anterior wall. The recirculation region adjacent to the leaflets completely loses its symmetry, evolving into a highly chaotic pattern. The jet becomes even narrower for them, while the asymmetric flow separation regions are considerably wider. In severe stenosis, the jet core intensifies and directly impacts the arch wall, exerting substantial force on the wall. Additionally, as stenosis severity increases, the vortex region at the posterior of the arch becomes more pronounced.

**Figure 5:**
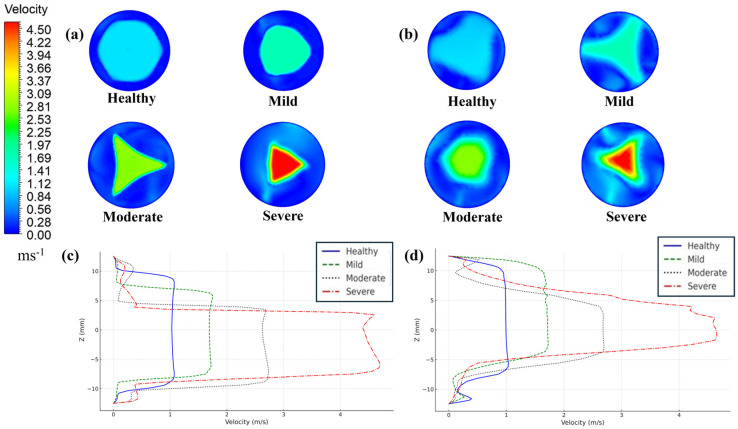
Cross-sectional velocity profile and distribution in the z-direction at the valve exit (**a**,**b**) and at 15 mm downstream (**c**,**d**).

Figure 5 indicates the effect of stenosis on the velocity profile in the aortic arch. Figure 5a,b represent the cross-sectional velocity distribution and velocity profile in the z-direction right at the jet exit, while Figure 5c,d portray their progression 15 mm downstream of the valve exit. Overall, the figure offers a comprehensive depiction of the velocity distribution progression within the ascending aorta. From Figure 5a, right at the exit, the flow pattern is uniform and wide for the healthy valve. However, as stenosis progresses, the jet narrows, like what is observed in mild stenosis, although the flow remains uniform. As stenosis severity increases, the flow distribution gets narrower and more skewed, taking the shape of the leaflet opening. This can be attributed to the high-velocity magnitude of the jet core. The skewness of the moderately and severely stenotic jets is more clearly observed in Figure 5b. It also illustrates the eccentricity of the recirculation domain for severe and moderate stenosis, which is located at the extremes of the z-direction. Severe stenosis exhibits a peak velocity of 4.7 m/s, significantly higher than the peak velocity of 2.5 m/s observed in moderate stenosis.

Additionally, as shown in Figure 5c, at 15 mm downstream, the jet core starts to dissipate in healthy valve and mild valve stenosis. However, the jet core persists at higher level of stenosis. Nonetheless, the exterior of the core interacts with the surrounding fluid and starts to lose its peak velocity gradually. No significant difference in the velocity magnitude is depicted across the models, as shown in Figure 5d. Furthermore, the profiles of severe and moderate stenosis lose some of their eccentricity, which becomes more pronounced in healthy and mild stenosis. No substantial difference in peak velocity is observed across any models in this case.

### 3.3. Evolution of Flow Structures Along the Aortic Arch

**Figure 6:**
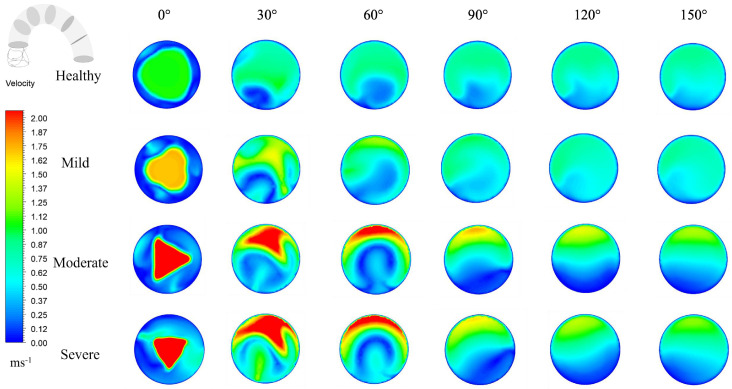
Progression of flow structure within the aortic arch.

To evaluate the progression of flow structure, the aortic arch has been divided into several cross sections at 30° intervals shown in Figure 6. Velocity contours have been generated for healthy valves, mild moderate and severely stenotic valves, respectively. From the figure, it is evident that a high-velocity jet emerges from the valve exit and gradually expands within the aortic arch. For moderate and severe stenosis, the jet remains narrow and concentrated along the centerline, whereas in healthy and moderate stenosis, the jet is much wider and more uniform. For the healthy valve, the overall velocity distribution remains uniform and symmetrical, and all the irregularities start to dissipate in the descending aorta when θ > 90°. Meanwhile, slight asymmetries and vortices are observed in the ascending aorta when θ < 60°. For mild stenosis, similar characteristics are observed in the aortic arch. The jet is much narrower and starts at a much higher speed, with a maximum velocity of 1.8 m/s. More irregularities are observed at lower θ, and the slight distortion starts to dissipate in a higher θ value gradually. However, the disturbance induced by stenosis remains evident in the flow domain for mild stenosis.

As stenosis severity increases, the flow becomes increasingly distorted and asymmetric. At θ = 0°, the flow profile takes on the shape defined by the leaflet opening, which is triangular for moderate and severe stenosis. Additionally, it is evident that the jet core in severe stenosis is much narrower than that in other conditions, indicating an increase in turbulence and flow separation. For both moderate and severe stenosis, the jet impinges on the arch wall at θ = 30°, causing the core to get disrupted. However, in the case of severe stenosis, the jet maintains a high velocity, indicating that for severe stenosis, the core region of the jet reaches up to the arch without losing significant energy. The presence of vortex and recirculation regions is apparent at higher θ, where flow predominantly moves along the anterior, while recirculation zones are located at the posterior. Interestingly, the velocity distribution and magnitude for moderate and severe stenosis become almost identical at θ > 90°, despite the jet initially having a considerably higher velocity in severe stenosis. This can be attributed to the fact that, in moderate stenosis, a significant portion of the jet core dissipates before reaching the arch wall. As a result, the flow follows the curvature of the arch more smoothly compared to severe stenosis, leading to minimal energy loss. In contrast, in the case of severe stenosis, the jet core impinges directly on the arch wall with most of its velocity, causing a substantial pressure drop and increased turbulence, particularly when θ = 30°, where a significant velocity reduction occurs. This substantial loss of kinetic energy in severe stenosis is further observed in its higher pressure loss coefficient.

### 3.4. Variation in Pressure Loss Coefficient

Dynamic pressure loss coefficient can be described as a dimensionless parameter that quantifies fluid resistance and energy loss in the flow domain with restriction while considering the flow velocity and fluid properties. The pressure loss coefficient (ξ) can be determined from the following equation [41]:

(4) $$\xi =\frac{\Delta P}{\frac{1}{2}\rho U^{2}}$$

where ΔP is the pressure drop (Pa) across the arch, ρ is the fluid density (kg/m^3^) and U is the velocity (m/s) in the left ventricle.

**Figure 7:**
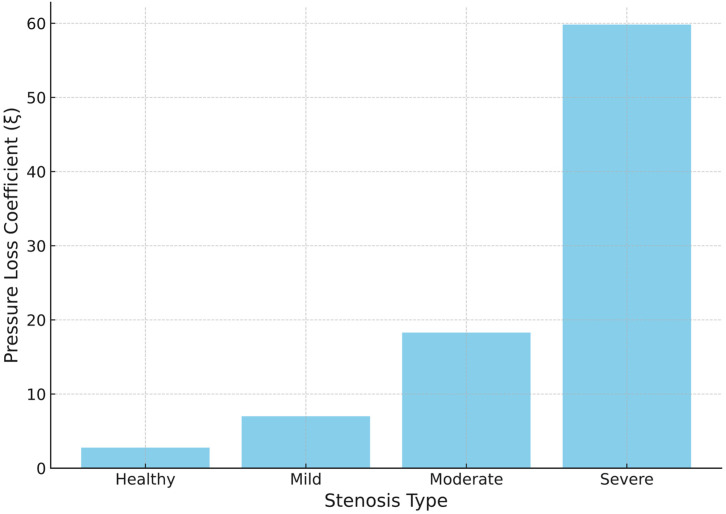
Pressure loss coefficient within the aortic arch for various severities of aortic stenosis.

As illustrated in Figure 7, the lowest ξ magnitude is observed by the healthy valve expectedly, indicating marginal loss of flow energy and uniformness of the velocity. The magnitude of ξ gradually increases with the level of stenosis and for moderate stenosis, ξ is approximately ten times greater than that in the healthy valve. However, severe stenosis indicates an even greater loss of energy, attaining a ξ three times greater than moderate stenosis. A significant portion of the coefficient can be attributed to the impingement of the high-velocity jet core on the wall, where it abruptly reaches a stagnation point and loses much of its kinetic energy. It explains why the velocity profile of moderate and severe stenosis becomes similar in the descending aorta. The observed pressure loss coefficient for healthy, mild, moderate, and severe stenosis are 2.78, 7.02, 18.33 and 59.96, respectively.

### 3.5. Changes in Helical Flow with Stenosis

**Figure 8:**
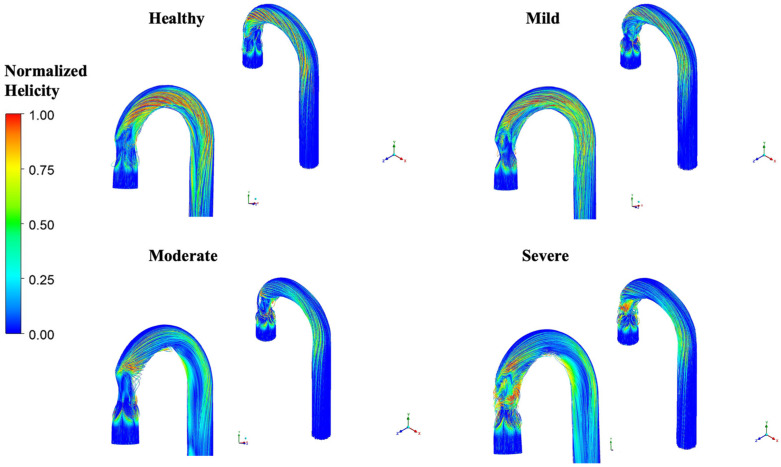
Normalized helicity across the domains for healthy, mild, moderate and severe stenosis.

Helicity indicates the intensity of helical flow within a domain, i.e., the cork-screw-like motion of fluids. It is calculated as the dot product of the velocity and vorticity fields. Normalized helicity is a dimensionless quantity, which is derived from the cosine of the angle between velocity and vorticity vectors [21]. In Figure 8, the normalized helicity is illustrated for various levels of stenosis. Absolute values have been taken for better presentation.

Figure 8 shows that the flow within the aortic arch exhibits significant helicity across all geometries, primarily due to the 180° bend. However, the intensity of helicity rises with the severity of stenosis, as observed in moderate and severe stenosis. It is also notable that the intensity of helicity is magnified in the ascending aorta, immediately downstream of the valve exit. This effect is pronounced in severe stenosis, as illustrated in the figure.

### 3.6. WSS Variation in the Aortic Arch and Valve Leaflets

**Figure 9:**
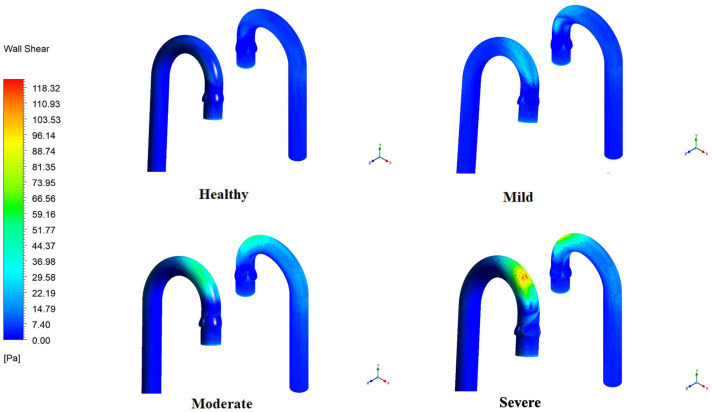
Distribution of WSS on the aortic arch.

Figure 9 illustrates the variation in WSS in the aorta at different severities of stenosis. The figure clearly shows that the peak WSS increases progressively with the severity of stenosis, increasing significantly at higher levels of stenosis. A higher WSS magnitude is observed at the anterior of the arch, where the jet emerging from the valve exit collapses. Subsequently, a peak WSS of 122 Pa is observed for severe stenosis, since the core jet region remains intact before impinging on the arch wall, resulting in a much higher and more non-uniform WSS distribution. The eccentricity of the jet plays a key role in both the magnitude and variation of WSS, as observed in moderate and severe stenosis. The peak magnitude for moderate stenosis in the arch is 80 Pa, followed by 35 Pa and 11 Pa for mild stenosis and healthy valves, respectively.

**Figure 10:**
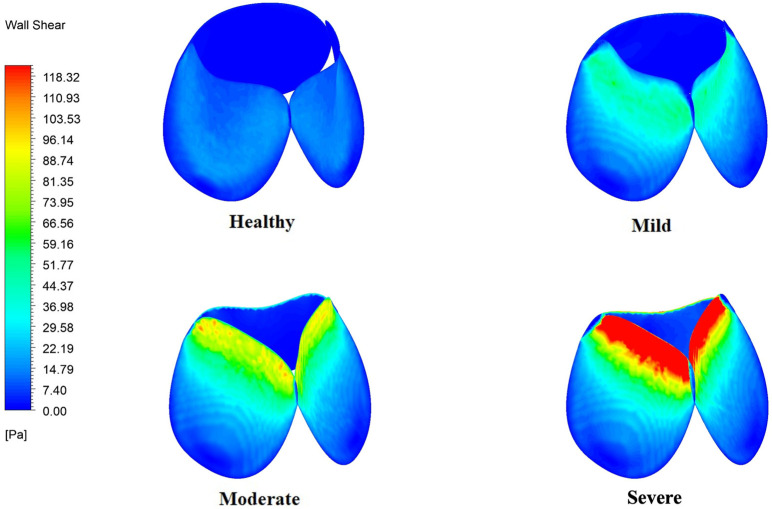
Variation of WSS in the leaflets, at different severities of stenosis.

Figure 10 shows the variation in WSS on the valve leaflets. The highest WSS intensity occurs at the tip of the leaflets, where the kinetic energy of the jet is the highest. The non-uniform WSS distribution along the sides of the leaflets can be attributed to recirculation zones adjacent to the leaflets. The asymmetrical vortical region in moderate and severe stenosis leads to a larger spread of high WSS along the sides of the leaflets. The maximum WSS values observed are 298 Pa, 122 Pa, 63 Pa and 30 Pa for severe, moderate, and mild stenosis, and healthy conditions, respectively.

## 4. Discussion

In this study, the hemodynamic abnormalities of mild, moderate, and severe valvular stenosis at peak systole have been compared with a healthy valve model using a steady-state CFD model. The hemodynamics of different stenosis models are characterized by velocity, pressure loss coefficient, helicity and WSS. A summary of the results can be found in Table 4.

### 4.1. Velocity Distribution and Maximum Velocity

The velocity in the aorta saw a sharp rise as the stenosis level increased, however, the change in peak velocity was not linear (R^2^ = 0.8). This non-linearity opposes the findings of Jhun et.al. (R^2^ = 0.98) [29], which can be attributed to the use of idealized valve leaflets in the current study, instead of an orifice to replicate the effective orifice area. Moreover, in the current study, as stenosis severity increases, the jet impinges on the arch wall and travels along the anterior of the arch. In contrast, the comparison study showed the jet with a relatively high velocity travelling through the posterior, likely due to the inclusion of valve leaflets in this investigation, and the difference in turbulence generation. A similar flow distribution was observed in other studies [38,42], although a uniform velocity was applied downstream of the valve in those instances as the inlet boundary condition. The velocity variation shown in Figure 4 aligns with previous studies [18], demonstrating a broad and homogeneous jet for healthy valves, and a constricted jet downstream for stenotic valves. These variations in the flow conditions of stenotic valves can lead to changes in the hemodynamic forces of the aorta such as the pressure gradients and shear stress.

The excessive hemodynamic forces exerted by the diseased valves include the possibility of valve and vessel wall remodeling. The extreme impingement velocity of severe stenosis, for example, can reduce the structural strength of the arch, which can eventually cause dilation of the aorta [7,8]. The flow field distribution presented in this study offers valuable insights for developing preliminary diagnostic guidelines for stenosis, potentially aiding in early intervention to prevent mortality.

### 4.2. Pressure Loss Coefficient

The pressure loss coefficient has shown its prospect as a parameter to differentiate the severity of aortic stenosis, as it accounts for both momentum and viscous losses, while not being influenced by heart rate [43]. A study conducted on 16 adult patients with mild to severe stenosis revealed a pressure loss coefficient between 9.2–57.4 based on Doppler-based pressure measurements, and between 10.5–54 for catheter-based pressure measurements [41]. In comparison, the present study calculated a pressure loss coefficient of 7.02 for mild stenosis, and 59.96 for severe stenosis, indicating the reliability of the current simulation. For a valve without stenosis, the coefficient was calculated to be 2.78. Therefore, the pressure loss coefficient can be considered a useful parameter for predicting the severity of stenosis, provided that further studies are conducted to establish a significant correlation between the coefficient and stenosis severity.

### 4.3. Helicity

It is evident from Figure 8 that helicity increases with the severity of stenosis, which can be linked with enhanced asymmetry and complexity of the flow in the aorta as stenosis progresses. Normalized helicity can be a crucial hemodynamic index to detect abnormalities in the aorta as it is associated with plaque deposition and aneurysm formation [44,45]. Additionally, the prolonged presence of abnormal helical flow structures may be associated with the beginning of hypertension [38,46]. A strong presence of helical flow indicates increased turbulence, which can lead to very high WSS [47], as supported by the results of the current investigation.

### 4.4. Wall Shear Stress

The simulation results indicate that WSS increases rapidly with the severity of stenosis and becomes more non-uniform. This trend is consistent with prior findings indicating that elevated and prolonged exposure to WSS impairs normal endothelial cell function, which can prompt vascular remodeling and lead to the development of various pathologies [12]. Moreover, increased WSS due to the presence of a jet with high-velocity magnitude and eccentricity can contribute to diseases in the ascending aorta, alongside the detriment of high molecular weight multimers (HMWM), which can result in gastrointestinal bleeding [29,48]. In the current study, the maximum WSS observed for severe stenosis on the arch is 122 Pa, while at leaflet it is 298 Pa. Researchers have linked WSS above 100 Pa to the onset of HMWM degradation, with complete disintegration occurring above 300 Pa, indicating a significant risk of HMWM destruction in severe stenosis [49]. Even in the case of moderate stenosis, there is a significant risk, as the maximum WSS reaches around 122 Pa in the vicinity of the valve leaflets. Since an eccentric valve jet impinges directly on the aortic wall, it delivers higher local kinetic energy and momentum flux, producing larger peak WSS and pressure-loss (ξ) than a centered jet.

### 4.5. Limitations

It is important to note that the current study focuses exclusively on the aortic hemodynamics at peak systole. However, to gain a further understanding of fluid dynamics, pulsatile flow conditions should be utilized. The highly dynamic motion of the leaflets can alter the flow condition significantly. To capture this behavior accurately, a FSI model should be developed in the following study. Additionally, the present model does not take the hyperelastic or viscoelastic properties of the vessels into consideration. The constant diameter of the ascending aorta and simplified arch geometry can limit the understanding of velocity gradient and turbulence. Moreover, since the hemodynamics within aorta are turbulent, to capture the fluctuations of turbulence parameters, higher order models such as LES (Large Eddy Simulation) and DNS (Direct Numerical Simulation) can be analyzed. It would allow to look at parameters such as RSS (Reynolds shear stress) for better understanding of the complex hemodynamics. To address these limitations, future studies should consider employing patient-specific geometry in the simulation to improve the physiological relevance.

## 5. Conclusions

In this study, the hemodynamic impacts of various severities of aortic stenosis in the aorta at peak systole was investigated using a 3D and steady-state CFD model. The velocity distribution, pressure loss coefficient, helicity and WSS were evaluated for mild, moderate, and severe stenosis and compared with a healthy baseline model, which provides detailed insight into the impact on the hemodynamic abnormalities of the aorta associated with increasing stenosis severity.

The accuracy and applicability of the numerical model were established by validating it against experimental data available in the literature. The results from this analysis demonstrated that with increasing stenosis severity, the jet velocity rises non-linearly and rapidly while increasing its eccentricity. The maximum velocity observed for the healthy, mild, moderate, and severe models were found to be 1.08, 1.82, 2.73 and 4.7 m/s, respectively. In addition, stenosis severity enlarged the recirculation region downstream adjacent to the leaflets. Also, the results reveal that severe stenosis leads to significantly higher WSS and pressure loss coefficient, contributing to turbulence generation that may exacerbate endothelial dysfunction and structural degradation of the aortic wall. Furthermore, the study underscores the importance of incorporating anatomically accurate leaflet geometries to correctly capture flow dynamics, highlighting the limitations of simplified orifice models.

## Figures and Tables

**Table 1:** Stenosis severity as categorized by the ACC/AHA [35].

Parameters	Mild	Moderate	Severe
Jet velocity (m/s)	<3.0	3.0–4.0	>4.0
Mean pressure gradient (mmHg)	<25	25–40	>40
Vascular area (cm^2^)	>1.5	1.0–1.5	<1.0

**Table 2:** Mesh sensitivity analysis of the computational domain.

Serial No.	Number of Cells (Millions)	WSS (Pa)	% Diff
1	0.7	2.55	5.73
2	1.2	2.411	2.15
3	2.5	2.361	0.59
4	4.4	2.347	-

**Table 3:** Validation of peak velocity magnitude against Jhun et al. [29] for moderate stenosis.

Parameters	Reference (Jhun et al.) [29]	This Study (CFD)	% Diff
Inlet flow rate	18 LPM	18 LPM	-
Orifice diameter	10 mm	10 mm	-
Peak centerline jet velocity [m/s]	4.20	4.12	1.9

**Table 4:** A quantitative summary of the results.

Metrics	Healthy	Mild	Moderate	Severe	%Δ vs. Healthy (Mild/Moderate/Severe)
Peak velocity in aortic arch [m/s]	1.08	1.82	2.73	4.70	+68.5%/+152.8%/+335.2%
Pressure-loss coefficient, ξ	2.78	7.02	18.33	59.96	+152.5%/+559.6%/+2057.0%
Pressure drop ΔP across arch [Pa]	323	815	2128	6962	+152.3%/+558.8%/+2055.4%
Arch WSS (peak) [Pa]	11	35	80	122	+218.2%/+627.3%/+1009.1%
Leaflet WSS (peak) [Pa]	30	63	122	298	+110.0%/+306.7%/+893.3%

## Data Availability

The original contributions presented in this study are included in this article. Further inquiries can be directed to the corresponding author.

## Abbreviations

The following abbreviations are used in this manuscript:

CFD	Computational fluid dynamics
WSS	Wall shear stress
3D	Three dimensional
4D	Four dimensional
MRI	Magnetic resonance imaging
PIV	Particle image velocimetry
LDV	Laser Doppler velocimetry
TKE	Turbulence kinetic energy
OSI	Oscillatory shear index
RSS	Reynolds shear stress
FSI	Fluid–structure interaction
2D	Two dimensional
